# Antimicrobial activity of two South African honeys produced from indigenous *Leucospermum cordifolium *and *Erica *species on selected micro-organisms

**DOI:** 10.1186/1472-6882-8-41

**Published:** 2008-07-15

**Authors:** Nicolaas J Basson, Sias R Grobler

**Affiliations:** 1 Oral and Dental Research Institute, Faculty of Dentistry, University of the Western Cape, Private Bag X1, Tygerberg 7505, South Africa

## Abstract

**Background:**

Honey has been shown to have wound healing properties which can be ascribed to its antimicrobial activity. The antimicrobial activity can be effective against a broad spectrum of bacterial species especially those of medical importance. It has also been shown that there is considerable variation in the antimicrobial potency of different types of honey, which is impossible to predict. With this in mind we tested the antimicrobial activity of honeys produced from plants grown in South Africa for their antibacterial properties on selected standard strains of oral micro-organisms.

**Methods:**

The honeys used were produced from the blossoms of *Eucalyptus cladocalyx *(Bluegum) trees, an indigenous South African plant *Leucospermum cordifolium *(Pincushion), a mixture of wild heather shrubs, mainly *Erica *species (Fynbos) and a *Leptospermum scoparium *(Manuka) honey. Only pure honey which had not been heated was used. The honeys were tested for their antimicrobial properties with a broth dilution method.

**Results:**

Although the honeys produced some inhibitory effect on the growth of the micro-organisms, no exceptionally high activity occurred in the South African honeys. The carbohydrate concentration plays a key role in the antimicrobial activity of the honeys above 25%. However, these honeys do contain other antimicrobial properties that are effective against certain bacterial species at concentrations well below the hypertonic sugar concentration. The yeast *C. albicans *was more resistant to the honeys than the bacteria. The species *S. anginosus *and *S. oralis *were more sensitive to the honeys than the other test bacteria.

**Conclusion:**

The honeys produced from indigenous wild flowers from South Africa had no exceptionally high activity that could afford medical grade status.

## Background

The potential of honey to assist with wound healing has been demonstrated repeatedly [1,2]. It has proven valuable in the treatment of infantile gastroenteritis, infected surgical wounds, burns, decubitus ulcers, skin crafting and surgical debridement [2-9]. The healing properties of honey can be ascribed to the fact that it offers antibacterial activity, maintains a moist wound environment that promotes healing and has a high viscosity which helps to provide a protective barrier to prevent infection [10].

It has been shown that natural unheated honey has some broad-spectrum antibacterial activity when tested against pathogenic bacteria, oral bacteria as well as food spoilage bacteria [10-12]. It is also clear from these studies that bacteria are not uniformly affected by honey. Furthermore, it has been shown that different honeys vary substantially in the potency of their antibacterial activity, which varies with the plant source [10,11,13,14]. At present a number of honeys are sold with standardized levels of antibacterial activity. The best known of these is New Zealand Manuka honey produced from the Manuka bush, *Leptospermum scoparium *[14].

The oral streptococci play an important role in oral health. They are involved in dental plaque development and the formation of dental caries. *Candida albicans *is an opportunistic pathogen of the oral cavity that may cause oral disease in especially the immune compromised individual, the elderly and those individuals wearing dentures. There is evidence that honey with a high antibacterial activity could be used to reduce dental plaque in the treatment of oral disease [15]. However little information is available with regards to effect of different honeys on putative oral pathogens.

The search for other honeys from different sources with enhanced antibacterial activity continues. South Africa has a large floral biodiversity with many unique plants indigenous to the region. Honeys are produced from these plants and sold commercially in South Africa. However none of these have been tested for their antimicrobial activity and possible use as medical grade honey. The aim of the present study was to investigate the antimicrobial activity of two South African honeys produced from indigenous plants unique to South Africa and two honeys produced from alien species against selected reference cultures of potential oral pathogens.

## Methods

The honey types used were as follows: a honey produced from the blossoms of *Leucospermum cordifolium*, a wild shrub unique to the Cape Peninsula of South Africa (Pincushion honey); a honey produced from a mixture of many heather shrubs but mainly from *Erica *species found on the southern coastal parts of South Africa (Fynbos honey); a honey produced from *Eucalyptus cladocalyx *trees (Bluegum honey) an alien species originally from Australia; a honey locally produced from the *L. scoparium *plant (Manuka bush) that was obtained from New Zealand. Honey types were identified and their source noted by the apiarists themselves. Only pure honey which had not been heated was used. The honeys were stored in the dark at room temperature. The honeys were tested for their antimicrobial properties against a control solution with sugar content similar to that of natural honey. The control carbohydrate solution contained 39% weight/volume (w/v) d-fructose, 31% (w/v) d-glucose, 8% (w/v) maltose, 3% (w/v) sucrose and 19% (w/v) water and was kept refrigerated when not in use [16].

The minimal inhibitory concentrations (MICs) were determined with a broth dilution method [17]. A 50% stock solution of honey and of the sugar control solution was prepared in double strength sterile Brain Heart Infusion (BHI) broth (Oxoid). Two-fold dilutions of the stock solutions were aseptically prepared in sterile BHI broth (Oxoid) using a carry-over technique to give a final volume of 5 ml in each tube. This was repeated until a dilution range of 50%, 25%, 12.5%, 6.25% and 3.12% of honey was obtained. After observing the range in which inhibition of growth in the honey solutions occurred, a closer dilution was prepared between dilutions of 25% and 12.5%.

Phenol was used as a positive control. A 40% stock solution was prepared in double strength BHI broth from an 80% phenol solution (Merck). Two-fold dilutions of the 40% dilution were aseptically prepared in BHI as described above.

The inocula used were prepared from overnight growth cultures in BHI-broth. One hundred microlitre of a 1/100 dilution of the culture was used to inoculate 5 ml of the test solutions. The tubes were incubated aerobically at 37 degrees C for 24 hours.

In view of the high risk of bacterial contamination from the environment, each honey sample was tested for sterility beforehand by inoculating 100 ml sterile BHI with 5 ml of the 50% stock solution. Furthermore, each growth positive tube in the tests was checked microscopically to confirm that the cell morphology corresponded with the cell morphology of the inoculum.

The microbial species tested were *Streptococcus mutans *(NCTC 10449), *Streptococcus salivarius *(NCTC 8618), *Streptococcus sanguis *(NCTC7864), *Streptococcus anginosus *(NCTC 10708), *Streptococcus gordonii *(NCTC 3165), *Streptococcus oralis *(NCTC 11427), *Streptococcus sobrinus *(NCTC 10921), *Candida albicans *(NCPF 3118), *Escherichia coli *(NCTC 9001) *and Staphylococcus aureus *(NCTC 8530).

The hydrogen peroxide concentration of each test dilution was determined one hour after dilution using Peroxid-Test strips (Merck). The pH of the undiluted honey, as well as the pH of each dilution was determined with a glass electrode. All tests were done in triplicate. The honey solutions were freshly prepared for each assay.

## Results

The minimum inhibitory concentrations (MICs) of the four natural honeys, including the control carbohydrate solution, are shown in Table 1. No difference was observed in the antimicrobial activity between the four honeys for concentrations up to 21%. At these concentrations, honey and the control solution had similar activity towards all organisms except for two streptococcus species. Both *S. anginosus *and *S. oralis *were inhibited by the honeys at concentrations of 17% and 12.5% respectively but grew up to a carbohydrate concentration of 21% (MIC 25%). At concentrations of 25%, the honeys and the control solution had variable activity towards the organisms tested. Bluegum, Pincushion and Manuka honey inhibited the growth of all test bacteria, but not the yeast. However, *E. coli *and *S. salivarius *grew in the Fynbos honey at this concentration. The carbohydrate control solution could not inhibit growth of *S mutans*, *S. salivarius*, *E. coli *and *C. albicans *at a concentration of 25%. No growth was observed at concentrations of 50% for the carbohydrate control or for the honeys tested.

**Table 1:** Minimum inhibitory concentrations (%) of different honeys towards standard strains of oral micro-organisms

**Organisms**	***E. cladocalyx *(Bluegum)**	***Erica species *(Fynbos)**	***L. cordifolium *(Pincushion)**	***L. scoparium *(Manuka)**	**Carbohydrate control**	**Phenol control**
*S. mutans *(10449)	25	25	25	25	50	<2.5
*S. salivarius *(8618)	25	50	25	25	50	<2.5
*S. sanguis *(7864)	25	25	25	25	25	<2.5
*S. anginosus *(10708)	17	17	17	17	25	<2.5
*S. gordonii *(3165)	25	25	25	25	25	<2.5
*S. oralis *(11427)	12.5	12.5	12.5	12.5	25	<2.5
*S. sobrinus *(10921)	25	25	25	25	25	<2.5
*C. albicans *(3118)	50	50	50	50	50	<2.5
*E. coli *(9001)	25	50	25	25	50	<2.5
*S. aureus *(8530)	25	25	25	25	25	<2.5

Hydrogen peroxide was detected only at dilutions of 6.25% and 3.12%, at a low concentration of 1 mg/liter. The pH of the undiluted natural honeys was in the order of ± 3.6. On dilution with the growth medium, the pH of each of the dilutions increased from ± 5.25 for the 50% dilution to ± 7.26 for the 3.12% dilution (Table 2).

**Table 2:** pH of the different honey solutions at each dilution

**Honey type**	**Dilution**						
	
	**50%**	**25%**	**21%**	**17%**	**12.50%**	**6.25%**	**3.12%**
*E. cladocalyx *(Bluegum)	5.25	6.41	6.47	6.69	7.10	7.20	7.28
*Erica *species (Fynbos)	5.19	6.28	6.39	6.54	6.71	6.96	7.15
*L. cordifolium *(Pincushion)	5.23	6.40	6.52	6.70	6.90	7.30	7.38
*L. scoparium *(Manuka)	5.17	6.30	6.45	6.64	6.89	7.18	7.30

## Discussion

Our study clearly shows that honeys produced from two indigenous South African flowers, the endemic *L. cordifolium *(Pincushion) and *Erica *spp. (Fynbos) do not have any exceptionally high broad-spectrum antimicrobial activity. Furthermore, these honeys were not any different from two alien species grown in South Africa. In previous studies, very similar results were obtained with *E. cladocalyx *(Bluegum) honey [12,18]. These honeys are commercially produced on a large scale and are easily obtainable off the shelf. However, on a very small scale, honeys are produced from other floral sources. Considering the large floral biodiversity in South Africa, a survey of these rare honeys might uncover a honey with high levels of antimicrobial activity.

The poor activity of Manuka honey was disappointing as previous reports have shown that Manuka honey may have a very high antimicrobial activity against a number of bacteria [13,19]. However, not all Manuka honey exhibits antibacterial activity. High antimicrobial activity is recorded only in Manuka honey produced from specific localities, in particular, the East Cape region of the North Island of New Zealand [20].

The results from this report confirm that honeys from different countries and regions have a wide variability in their antimicrobial activity. It has been shown that the antimicrobial activity of honey may range from concentrations lower than 3% to concentrations of 50% and higher [10,13,19]. The honeys we tested had antimicrobial activity in the 17 – 25% range against the organisms that were tested except for *S. oralis *which showed sensitivity at 12.5%. The bacteria *S. aureus *and *E. coli*, which were included as reference cultures to previous reports, were both inhibited in the 50 – 25% range. These species have been shown to be sensitive at concentrations as low 1.8 – 11% for some honeys [13,21]. This indicates that our honeys were of a poor antimicrobial quality.

Honey may inhibit bacterial growth due to a number of different reasons. High sugar concentration (reduced water activity), low pH, hydrogen peroxide generation, proteinaceous compounds or other unidentified components present in the honey may all provide antimicrobial activity [11]. Low pH can be ruled out in this study because the average pH of the honey samples at 21% was 6.47 which falls within the growth range of the micro-organisms. Although hydrogen peroxide was produced in our study, it only occurred at the very low dilutions and at concentrations of ± 1 mg/litre. These concentrations are too low to have any bacteriostatic effect. Concentrations of at least 10 mg/litre are required to inhibit bacterial growth [22]. It is however possible that the hydrogen peroxide production was not at its peak at the time of testing. It has been shown that hydrogen peroxide production can peak at different times for different honeys. Some may take as long as 24 hours [23]. If bacteriostatic concentrations were obtained within the 24 hour incubation period, lower MICs would probably have been attained.

The honey MICs were very similar to the carbohydrate control solution which suggests that the main inhibitory effect was probably as a result of the reduced water activity, the only exceptions being the bacteria *S. anginosis *and *S. oralis*. Both these strains were sensitive to honey at concentrations lower than that observed for the carbohydrate control, indicating that an unidentified component, that is active against these organisms, may be present in the honey.

## Conclusion

We conclude that the honeys produced from the two endemic wildflower plants from South Africa do not differ from the alien species tested and show no exceptionally high antimicrobial activity that could provide medical grade status.

## Competing interests

The authors declare that they have no competing interests.

## Authors' contributions

NJB participated in the design of the study, carried out the microbiological analysis and drafted the manuscript. SRG conceived the study, participated in its design, was responsible for collection and identification of the honey samples and assisted in drafting the manuscript. All authors read and approved the final manuscript.

## Pre-publication history

The pre-publication history for this paper can be accessed here:


